# Supplementary material for: Genome-Wide Association Study of Resistance to Largemouth Bass Ranavirus (LMBV) in Micropterus salmoides
Source: Int J Mol Sci. 2024 Sep 18;25(18):10036. doi: 10.3390/ijms251810036 (PMC11432711; doi:10.3390/ijms251810036)
Supplement: Supplementary file 1 [file ijms-25-10036-s001.zip › ijms-3135708-supplementary.pdf]

Table S1: SNP-InDel location information statistics.

Variation type	Number	Type	Number
SNP	1925	downstream	52
		nonsynonymous SNV	32
		synonymous SNV	58
		intergenic	693
		intronic	905
		ncRNA_exonic	4
		ncRNA_intronic	18
		upstream	52
		upstream;downstream	6
		UTR3	64
		UTR5	41
Indel	423	downstream	14
		exonic	4
		intergenic	148
		intronic	208
		ncRNA_exonic	1
		ncRNA_intronic	10
		splicing	1
		upstream	15
		upstream;downstream	4
		UTR3	9
		UTR5	9
